# Prevalence and correlates of complementary and alternative medicine use among diabetic patients in Beirut, Lebanon: a cross-sectional study

**DOI:** 10.1186/1472-6882-14-185

**Published:** 2014-06-06

**Authors:** Farah Naja, Dana Mousa, Mohamad Alameddine, Hikma Shoaib, Leila Itani, Yara Mourad

**Affiliations:** 1 Faculty of Agriculture and Food Sciences, Department of Nutrition and Food Sciences, American University of Beirut, Riad El-Solh, Beirut 1107 2020, Lebanon; 2 Faculty of Health Sciences, Department of Health Management and Policy, American University of Beirut, Riad El-Solh, Beirut 1107 2020, Lebanon; 3 Faculty of Health Sciences, Department of Nutrition and Dietetics, Beirut Arab University, Riad El-Solh, Beirut 1107 2809, Lebanon

**Keywords:** Complementary and alternative medicine, Type-two diabetes mellitus, Prevalence, Patient safety, Integration, Policy, Lebanon

## Abstract

**Background:**

Patients with Type 2 Diabetes Mellitus (T2DM) are increasingly using complementary and alternative medicine (CAM) therapies due to difficulty in adhering to the therapeutic regimens and lifestyle changes necessary for disease management. Little is known about the prevalence and mode of CAM use among patients with T2DM in Lebanon.

**Objective:**

To assess the prevalence and modes of CAM use among patients with T2DM residing in Beirut, Lebanon.

**Methods:**

A cross-sectional survey of T2DM patients was conducted on patients recruited from two major referral centers in Beirut- a public hospital and a private academic medical center. In a face-to-face interview, participants completed a questionnaire comprised of three sections: socio-demographic, diabetes characteristics and types and modes of CAM use. Descriptive statistics, univariate and multivariate logistic regression analyses were utilized to assess the prevalence and correlates of CAM use, as well as whether the use was complementary or alternative to mainstream medicine. The main outcome in this study, CAM use, was defined as using CAM at least once since diagnosis with T2DM.

**Results:**

A total of 333 T2DM patients completed the survey (response rate: 94.6%). Prevalence of CAM use since diagnosis with the disease was 38%. After adjustment, CAM use was significantly associated with a “married” status, a longer duration of T2DM, the presence of disease complications, and a positive family history of the disease. Folk foods and herbs were the most commonly used CAM followed by natural health products. One in five patients used CAM as alternative to conventional treatment. Only 7% of CAM users disclosed the CAM use to their treating physician. Health care practitioners were the least cited (7%) as influencing the choice of CAM among users.

**Conclusion:**

The use of CAM therapies among T2DM patients in Lebanon is prevalent. Decision makers and care providers must fully understand the potential risks and benefits of CAM therapies to appropriately advise their patients. Attention must be dedicated to educating T2DM patients on the importance of disclosing CAM use to their physicians especially patients with a family history of diabetes, and those who have had the disease for a long time.

## Background

Diabetes is a chronic debilitating medical condition that, despite recent advances in care and management, still precipitates substantial morbidity, mortality and long term complications on patients and their families [1]. The global prevalence of diabetes is estimated to reach 7.7% by 2030 [2]. Over the past decade, several epidemiological studies in the Eastern Mediterranean Region (EMR) have reported high prevalence of Type-2 Diabetes Mellitus (T2DM) and impaired glucose intolerance [1,3-5]. In fact, the EMR has a diabetes prevalence that is among the highest in the world with an estimated 9.2% of the adult population having the disease [5]. The prevalence of T2DM among adults in Lebanon (15.8%) is lower than that reported in many other EMR countries, including Bahrain (25.5%), UAE (23.3%) and KSA (23.7%) [6,7]. Yet, researchers are alarmed by the increasing trend in prevalence of T2DM over time, since this prevalence in the country has increased by 36% in a five-year period, from 11.6% in 1999 to 15.8% in 2004 [6,8].

Although the use of insulin is considered to be a quantum leap in the management of diabetes, living with T2DM remains a challenge requiring considerable dedication and commitment to a life-long regimen imposed by this chronic disease [9]. In addition, the achievement of good T2DM control is often difficult due to the required lifestyle changes, including: modifying eating habits, maintaining optimal body weight, exercising regularly and self-monitoring of blood sugar [10]. Non-compliance with long term management of T2DM may lead to serious negative effects on health systems, such as compromised health benefits and serious economic consequences in terms of wasted time, money and uncured disease [11].

As a result of the chronic course of the disease, the debilitation of complications and the complexities of treatment plans, many T2DM patients manage their disease through the use of complementary and alternative medicine (CAM) therapies [10]. The US National Center for CAM therapies divides CAM into four categories: (1) Mind-body systems; (2) Manipulative and body-based practices; (3) Energy Medicine; and (4) Biologically based practices [12]. In this manuscript, CAM refers to biologically based practices including substances found in nature, such as herbs, dietary supplements, multivitamin and mineral supplements, as well as prayers. Such therapies are used for the prevention and treatment of diseases and are meant to complement mainstream medicine by: “satisfying a demand not met by orthodoxy or by diversifying the conceptual frameworks of medicine” [13]. CAM therapies are gaining public acceptance and are increasingly used around the globe, especially among individuals with chronic illnesses such as T2DM [14-16]. Patients may resort to CAM use for a multitude of reasons including: dissatisfaction with conventional treatment, the adverse effects experienced with drugs and the high cost of such drugs [17-20]. Additional reasons include patients’ need to have personal control over the course of their disease, as well as the perceived compatibility of CAM therapies with patients’ values, spiritual/religious philosophy and beliefs regarding the nature and the meaning of death and illness [17,19-22].

However, despite the growing popularity of CAM use, there is still insufficient evidence to draw conclusions about the efficiency of many common CAM therapies, including herbs and supplements for prevention and management of diabetes [23]. While several comprehensive reviews have found evidence on the effective use of extracts of plants in the treatment of diabetes [24,25], few studies reported significant side effects of CAM use in T2DM [26].

In the EMR, little is known about the prevalence of use of CAM therapies in general and among diabetic patients in particular. High rates of CAM use, extending from 25% to 85% of individuals with diabetes, were reported in Turkey [27-29]. In Bahrain the prevalence rate was also high among diabetic patients, estimated at 63%, whereas in Jordan it was noted to be 16.6% [4,30]. Notwithstanding the fact that Lebanon is a country where CAM use is both prevalent and culturally accepted, there is no available data on the prevalence and determinants of CAM use among Lebanese diabetic patients. Investigating the prevalence of CAM use, the causes and modes for use, and patients disclosure of such use is crucial since results could help protect the health of patients, improve the patient-provider communication and coordination, and help integrate CAM therapies into mainstream medicine.

The aim of this study is to investigate the prevalence and socio-demographic correlates of CAM use among T2DM patients in Lebanon and to characterize the mode of CAM use in this patient population, whether CAM was used as complementary or alternative to mainstream medicine. Such investigation aims at providing some essential evidence that could guide decision making at the system, institutional and individual levels.

## Methods

A cross-sectional study assessing the prevalence, types, modes and determinants of CAM use among Lebanese T2DM patients was conducted between the months of August 2010 and January 2011. The subjects were from the two major referral centers in Beirut- a public hospital and a private academic medical center. Both medical centers are accredited by the Lebanese Ministry of Health and attract a large proportion of the patient population in the Greater Beirut Area. Ethical approval for this study was obtained from the Institutional Review Board (IRB) of the Social and Behavioral Sciences at the American University of Beirut, under protocol number NUT.FN.04.

### Subjects and procedure

The pool of participants in this study included patients older than 18 years of age of both genders who had been diagnosed with T2DM for at least one year prior to recruitment and who have returned to either of the two hospitals for continuation of the treatment. For sample size calculation, the following formula was used [31]: n = Z^2^_1 ‒ α/2_P(1 ‒ P)/e2.

Where

n = number to sample

Z^2^ = (1.96)^2^ for 95% confidence

P = “best guess” for prevalence

e = maximum tolerable error for the prevalence estimate

Assuming a 35% prevalence of CAM use among T2DM (P), a 95% confidence interval (Z = 1.96) and a prevalence estimate within 5% error margin (e), a sample of 344 adults with T2DM was deemed appropriate for this study.

With the consent of target institutions and treating physicians, all patients fitting the inclusion criteria were recruited while waiting for their turn in the clinics waiting areas on random days. The nurses in charge at the clinics introduced the study to all eligible patients present in the waiting areas. A nurse and a graduate student in Nutritional Sciences carried out data collection. Both field workers had undergone extensive training in questionnaire administration and interviewing techniques and were clearly instructed and reminded to avoid asking any leading questions that could bias patients’ answers in any particular direction. In addition, weekly meetings of the research team were held to ensure the inter-rater reliability and the standardization of data collection protocol. Each participant was interviewed only once. Health care providers responsible for the treatment of interviewed patients were not present during the interviews. The participants were assured that any information they reveal would remain confidential and would be strictly used for research purposes only. Patients were not paid to take part in the study and were informed that they were free to decline answering any questions they were not comfortable with. Signed consent forms were obtained from all participating patients. Each interviewed patient was administered a survey questionnaire, filled out through a face-to-face interview. Interviews took an average of 20 minutes to complete. To ensure a representative cross-sectional sample of patients from the two participating medical centers, interviews were conducted on different days of the week and at varying times.

### Survey instrument

The survey instrument was divided into three sections: socio-demographic characteristics, diabetes-related characteristics and modes and experience of CAM use. Socio-demographic data collected included: age, sex, place of birth, marital status, educational level, employment status, health insurance and monthly income. The second section of the questionnaire included questions related to T2DM, such as: age at diagnosis, duration of the disease, family history of T2DM, and presence of T2DM complications. The last part of the questionnaire focused on CAM use. This section included questions about the type of CAM used, factors influencing the decision to use CAM, the reasons for CAM use, experience of any side effect as a result of the CAM used, the use of CAM as alternative or complementary to main stream medicine, the disclosure of CAM use to treating physicians and the reasons for not using CAM.

The content validity of the survey instrument was confirmed by an expert panel consisting of a physician, a nutrition epidemiologist and a health management and policy expert. Except for the question about the religious beliefs of the patients, all of the questions included in the survey instrument were deemed appropriate. Given the political tension in the country among various religious parties, the panel decided to drop this question despite the suggested influence of such beliefs on the use of CAM modalities. The original version of the questionnaire was written in English and subsequently translated to Arabic (since the majority of patients spoke Arabic). The translated Arabic version was back translated by a professional translator to ensure the parallel-form reliability of the questionnaire. The original and the back translated versions were reviewed for consistency in meaning by two bilingual experts. A pilot study was conducted with 15 selected diabetic patients to ensure that the target population understood the questions and that the answers yielded the required data. The findings of the pilot study were included in the analysis of the data for the present study.

### Statistical analysis

The data was checked for completeness, and responses were coded and entered into the Statistical Package for the Social Sciences (SPSS) software version 18 for Windows, which was later used for statistical analyses [32]. Frequencies and percentages were used to assess the prevalence, types, mode and patterns of CAM. CAM use, the main outcome in this study, was defined as using CAM at least once since diagnosis with T2DM. Chi-square and independent t-tests were used to chart comparisons of categorical and continuous variables between users and non-users of CAM. Univariate and multivariate logistic regression analyses were applied to determine which factors are associated with the use of CAM. In the regression model, CAM use was used as the dependent variable. In addition to age and sex, characteristics that showed statistical significance in the univariate analysis were included in the multivariate model as independent variables. Odds ratios and their respective 95% confidence intervals were calculated. A p-value of 0.05 was used to determine statistical significance.

## Results

Out of 352 T2DM patients invited to participate in this study, a total of 333 completed the survey questionnaire (response rate 94.6%). The main reasons for refusal to participate were lack of time or disinterest in the study objectives. Table 1 displays the socio-demographic and disease-related characteristics of the overall study population including CAM users and CAM non-users. Comparable proportions of patients were recruited from the private academic medical center and the public hospital (46.2% vs 53.8%). Patients’ average age was 60.29 ± 11.89 years, with 184 (55.3%) males and 238 (71.5%) married. The sample population comprised subjects from all levels of education ranging from illiterate (11.8%) to university level (26%). A considerable proportion of patients (56.3%) reported no health insurance coverage and about 41% reported a monthly income less than 500$. The average age at diagnosis was 49.1 ± 12.27 years. More than half of the study population reported a positive family history of T2DM (54.7%) and the presence of complications as a result of their T2DM (58.6%). Compared to the private academic medical center, patients attending clinics in the public hospital were more likely to use CAM (OR: 1.57; CI: 1.002-2.46). Using bivariate logistic regression, factors that were associated with CAM use in the study population included site of recruitment, marital status, monthly income, duration of T2DM, family history of the disease, as well as presence of T2DM complications. Compared to not married, married patients had twice the odds of using CAM (OR: 1.96; CI: 1.16-3.29). Patients reporting an income less than 500$ or more than 1000$ had significantly lower odds of using CAM as compared to those reporting middle income range (500-100$) (OR: 0.45; CI: 0.24-0.86 and OR: 0.44; CI: 0.23-0.85 respectively). The longer the duration of diabetes the higher were the odds of CAM use with patients diagnosed for a period over 15 years having the highest odds (OR:3.37; CI: 1.56-7.29). “Having a positive family history of diabetes” and “presence of diabetes complications” were both associated with higher odds of CAM use (OR: 1.25; CI: 1.10-1.38 and OR: 1.58; CI: 1.003-2.50 respectively).

**Table 1:** Socio-demographic and disease-related characteristics of the study population (n = 333) and their association with CAM use

**Characteristic**	**Overall (n = 333)**	**CAM users (n = 127)**	**CAM non-users (n = 206)**	**OR (95% CI)**
Site/Medical Center (n = 333)				
AUB-MC	154(46.2)	50(32.5)	104(67.5)	1
RHUH	179(53.8)	77(43.0)	102(57.0)	1.57(1.00-2.46)
Age (years) (mean ± SD) (n = 333)	60.20 ± 11.89	58.52 ± 10.98	61.20 ± 12.33	0.98(0.96-1.00)
Sex (n = 333)				
Male	184(55.3)	76(41.3)	108(58.7)	1
Female	149(44.7)	51(34.2)	98(65.8)	0.74(0.47-1.16)
Place of birth (n = 333)				
Village	95(28.5)	42(44.2)	53(55.8)	1
Town-city	238(71.5)	85(35.7)	153(64.3)	0.70(0.43-1.14)
Marital status (n = 333)				
Not married	95(28.5)	26(27.4)	69(72.6)	1
Married	238(71.5)	101(42.4)	137(57.6)	1.96(1.16-3.29)
Education (n = 331)				
Illiterate	39(11.8)	15(38.5)	24(61.5)	1
Elementary school	119(36.0)	53(44.5)	66(55.5)	1.28(0.613-2.69)
High school	87(26.3)	31(35.6)	56(64.4)	0.89(0.41-1.93)
University level	86(26.0)	26(30.2)	60(69.8)	0.69(0.31-1.53)
Employment (n = 333)				
Not employed	221(66.4)	83(37.6)	138(62.4)	1
Employed	112(33.6)	44(39.3)	68(60.7)	1.08(0.67-1.72)
Presence of health insurance (n = 332)				
Uninsured	187(56.3)	79(42.2)	108(57.8)	1
Insured	145(43.7)	48(33.1)	97(66.9)	0.68(0.43-1.06)
Monthly income (n = 286)				
500-1000$	59(20.6)	32(54.2)	27(45.8)	1
<500$	117(40.9)	41(35.0)	76(65.0)	0.45(0.24-0.86)
>1000$	110(38.5)	38(34.5)	72(65.5)	0.44(0.23-0.85)
**Diabetes related characteristics**				
Age at diagnosis of T2DM (years) (mean ± SD) (n = 333)	49.09 ± 12.27	46.28 ± 10.546	50.83 ± 12.940	0.97(0.95-1.01)
Duration of T2DM (years) (mean ± SD) (n = 333)				
1-2 years	59(17.7)	12(20.3)	47(79.7)	1
3-5 years	52(15.6)	22(42.3)	30(57.7)	2.87(1.24-6.65)
6-10 years	79(23.7)	34(43.0)	45(57.0)	2.96(1.36-6.42)
11-15 years	63(18.9)	22(34.9)	41(65.1)	2.10(0.93-4.76)
>15 years	80(24.0)	37(46.3)	43(53.8)	3.37(1.56-7.29)
Family history of diabetes mellitus (n = 333)				
No	151(45.3)	53(35.1)	98(64.9)	1
Yes	182(54.7)	74(40.7)	108(59.3)	1.25(1.10-1.38)
Presence of diabetes complications (n = 333)				
No	138(41.4%)	44(31.9)	94(68.1)	1
Yes	195(58.6%)	83(42.6)	112(57.4)	1.58(1.003-2.50)

A multivariate logistic regression model was used to examine the correlates of CAM use in the study population (Table 2). Variables were put in the model in order of strength of their association with CAM use as per the bivariate analysis. The effect of each variable on the model was assessed and the variable was kept if it significantly contributed to a better fit of the model. The final model included the following variables: site of recruitment, age, sex, marital status, education monthly income, duration of T2DM, presence of diabetes complications, and family history of diabetes. The results of the multivariate model showed that CAM use was significantly associated with a “married” status (OR 1.95; 95% CI: 1.02-3.84), a longer duration of T2DM (OR 2.99; 95% CI: 1.23-7.28 for patients diagnosed with T2DM longer than 15 years as compared to 1–2 years), the presence of disease complications (OR: 1.71; 95% CI: 1.3-3.51), and a positive family history of the disease (OR: 1.15; 95% CI: 1.02-1.30).

**Table 2:** Correlates of CAM use using multivariate logistic regression

**Characteristic**	**OR (95% CI)**
Site/Medical Center (n = 333)	
AUB-MC	1
RHUH	1.44(0.60-3.44)
Age (years) (mean ± SD) (n = 333)	0.98(0.96-1.004)
Sex (n = 333)	
Male	1
Female	0.83(0.44-1.59)
Marital status (n = 333)	
Not married	1
Married	1.95(1.02-3.84)
Education (n = 331)	
Illiterate	1
Elementary school	0.73(0.29-1.85)
High school	0.60(0.20-1.77)
University levels	0.67(0.19-2.44)
Monthly income (n = 286)	
500-1000$	1
<500$	0.48(0.23-1.02)
>1000$	0.67(0.28-1.61)
Duration of T2DM (years) (mean ± SD) (n = 333)	
1-2 years	1
3-5 years	2.06(0.80-5.32)
6-10 years	2.65(1.14-6.17)
11-15 years	1.60(0.64-3.98)
>15 years	2.99(1.23-7.28)
Presence of diabetes complications (n = 333)	
No	1
Yes	1.71(1.3-3.51)
Family history of diabetes mellitus (n = 333)	
No	1
Yes	1.15(1.02-1.30)

Table 3 describes the prevalence, modes and characteristics of CAM use among study participants. The prevalence of CAM use in the study population was 38%, 95% CI (33.1-43.5). Seventy patients (21%) have used CAM at least once during the last 12 months. The majority of CAM users (66.1%) reported that their choice of the CAM therapy was influenced by their friends while only 7.1% were guided by health practitioners. Trying for the sake of experimentation and believing in the advantages of CAM practices were the most commonly cited reasons for CAM use (63.8% and 41.7% respectively). Forty five patients (35.4%) described the CAM they have used as “not all useful”; thirteen patients (10.2%) reported experiencing side effects due to CAM they used, and almost one in two CAM users (46.5%) said that they will not use it again.

**Table 3:** Prevalence, modes and characteristics of CAM use among patients with T2D (n = 333)

**Prevalence and types of CAM used among diabetic patients**	**n (%)**
Used CAM in the previous year	
Yes	70(21)
No	263(79)
Used CAM since diagnosis	
Yes	127(38.1)
No	206(61.9)
**CAM related characteristics among CAM users (n = 127)**	
CAM choice*	
Friends	84(66.1)
Media	25(19.7)
Family believes	14(11.0)
Personal choice	13(10.2)
Health practitioner	9(7.1)
Reasons of CAM use*	
Trying CAM for the sake of experiment	81(63.8)
Belief in advantages of CAM practices	53(41.7)
Looking for another solution	23(18.1)
Lost hope with conventional therapy	6(4.7)
CAM is accessible and available	1(0.8)
What was your expectation when you were using CAM	
Prevent progression of diabetes	34(26.8)
No expectations	29(22.8)
Complete cure of disease	28(22.0)
Lowering blood glucose level	24(19.0)
Weight loss	3(2.4)
Better health status	2(1.6)
Other**	6(4.7)
Feeling after CAM use*	
Feeling of strengthening of body	60(47.2)
Feeling of no change	58(45.7)
Feeling of disappearance of several symptoms	40(31.5)
Feeling of being in good psychological condition	31(24.4)
Feeling physically worse	7(5.5)
Feeling rise of several symptoms	6(4.7)
Not decided	2(1.6)
Feeling of being in bad psychological condition	1(0.8)
Fear of the product and its effect	1(0.8)
Improvement of sexual life	1(0.8)
How do you assess the usefulness of CAM?	
No useful at all	45(35.4)
Very useful	30(23.6)
Not sure/unable to assess	28(22.0)
Of limited usefulness	24(18.9)
Side effects from CAM use	
Yes	13(10.2)
No	113(89.0)
Undecided	1(0.8)
Would you use CAM again?	
Yes	64(50.4)
No	59(46.5)
Undecided	4(3.1)
Would you Recommended CAM to other T2DM patients?	
Yes	46(37.1)
No	49(39.5)
Undecided	29(23.4)
Was your use complementary or alternative (n = 86)?	
Complementary	68(79.1)
Alternative	18(20.9)
Did you consult a doctor before using CAM (n = 130)?	
Yes	9(6.9)
No	121(93.1)
**CAM related characteristics among diabetic non-users (n = 206)**^†^	
Reasons for not using CAM*	
Do not believe in it	133(64.6)
The doctor did not prescribe it	58(28.1)
Afraid of the side effects	47(22.8)
Never heard of it	15(7.3)
Additional expenses and useless	14(6.8)
Do no need it	12(5.8)
Mainstream medicine is the best	4(1.9)
Not interested	4(1.9)
CAM is not evidence based	3(1.4)
No one advised its use	3(1.4)
Other***	3 (1.4)
Would you consider using CAM in the future	
Yes	37(18.0)
No	169(82.0)

*More than one answer was applicable.

Other **: *Other expectations when you were using CAM: Getting rid from sorcery spell (n = 1), Cure from osteoarthritis (n = 1), Pain relief (n = 2), Stop using medications (n = 1), feeling psychologically better (n = 1).

Other ***There is no definite and ultimate cure for T2DM, Negligence, Old way of treating diseases.

^†^These questions were asked only to CAM non users.

Eighteen patients (20.9%) used CAM as alternative to the mainstream medicine while 79.1% used the CAM on complementary basis. The comparison of socio-demographic and disease related characteristics between patients who used CAM as complementary and those who used it as alternative to main stream medicine showed no differences except for health insurance whereby a significantly higher proportion of alternative use was observed among un-insured compared to insured patients (27.3% vs. 6%, Chi-Square: 19.7; p < 0.05 respectively) (data not shown).

**Figure 1:**
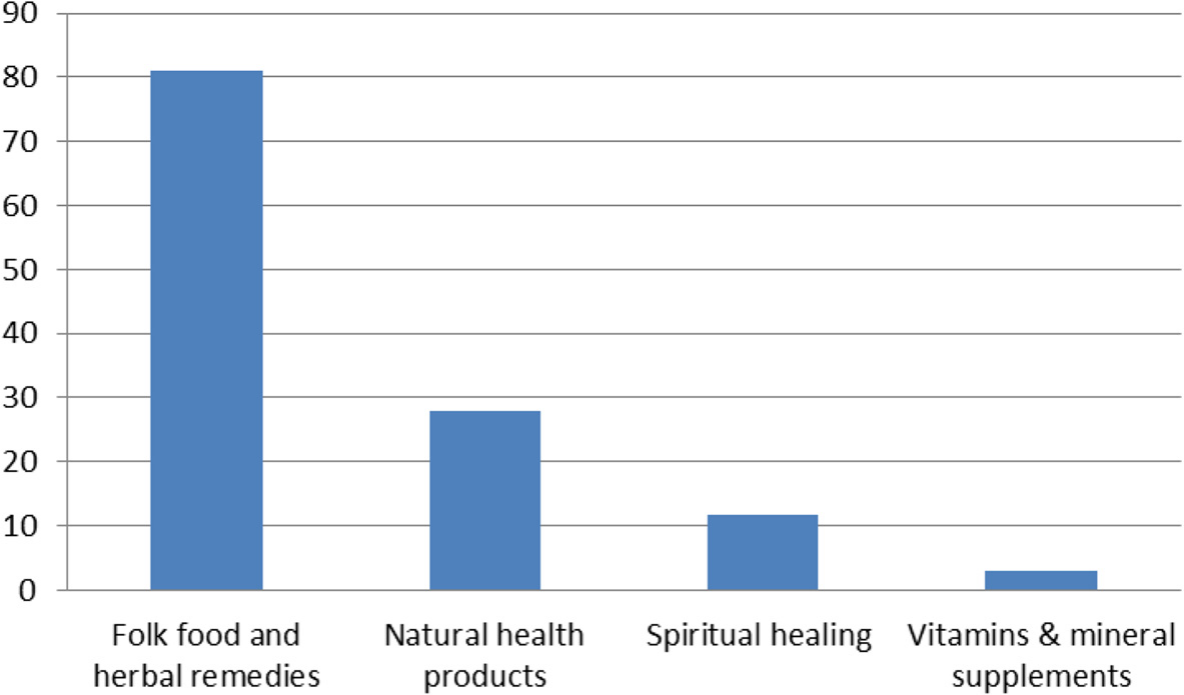
Distribution (%) of various CAM types as used by the study population^*^.

Only 38.9% of CAM users discussed the CAM modality they were using with a physician.Folk food and herbal remedies were the most common type of CAM used (81%) among study participants (Figure 1). Other CAM modalities used by the study population were natural health products (28%), spiritual healing (11.8%), and vitamins and minerals (3%) (Figure 1). Folk food and herbal remedies included functional foods and herbal infusions that belonged to the Lebanese folk methods of healing and were available in traditional herbal stores. Natural health products referred to prepackaged natural products that presented a health claim. Most of the Natural health products used by the study population were produced locally (90%) while the rest were imported. Spiritual healing included mainly prayers and vows. In the category of “vitamins and minerals”, multivitamins preparations and vitamin C were reported.

## Discussion

The study revealed that 38.1% of surveyed patients attending diabetes clinics in Beirut reported at least one incidence of using CAM therapies since diagnosis with T2DM. Other studies have reported a varying range of CAM use rates among T2DM patients depending on the country or geographic region. Compared to our findings, studies from Europe and North America yielded lower prevalence estimates. For example, among diabetes patients attending outpatient’s clinics in the UK, 17% reported the use of CAM [33]. In Canada, 25% of patients with diabetes used a type of CAM therapy [34]. Conversely, prevalence estimates of CAM use among T2DM patients were higher in South America and Asia as compared to our findings. For instance, the prevalence of CAM use among T2DM patients in Mexico was estimated at 62% [35]. In Asia, the prevalence estimates of 61%, 67.7% and 65% were observed in Taiwan, India and Korea respectively [36,37]. In Malaysia, a cross sectional study investigating the use of CAM among T2DM in the primary care setting found a prevalence of use of 62.5%, , similar to other rates observed in Asia [38]. In the Middle East region, studies among Jordanian and Palestinian patients found prevalence of CAM use of 16.6% and 51.9% respectively[4,39]. These observed variations in CAM use by geographic region could be in part attributed to differences in socio-cultural perceptions of CAM use and to disparities in the availability and access to conventional medicine. In addition, differences in study designs and definitions of CAM might have also contributed to the varying prevalence estimates of CAM use by T2DM patients in these countries [7].

Study findings revealed that folk foods and herbs were the most commonly used CAM followed by natural health products, spiritual healing and vitamins and minerals supplements. Lebanon is a small country in a region earlier referred to as “Bilad al Sham” – the Levant. This region was endowed with a high floral diversity that has for long constituted a basis for health care with very few species imported from outside it [40]. Lebanese herbalists, similar to other Arab herbalists in the region, have managed to maintain relics of the traditions alive into the twenty-first century and they still include, in their repertoire of medicinal use, hundreds of plant species even though very few of these plants have had their medicinal properties investigated using contemporary evidence based medicine [41,42]. Our results showed that, the cultural value of herbal treatments remains part of the collective memory of the people of the region despite its marginalization and decline. In addition, ease of accessibility and lower cost of folk products may have contributed to the observed wide spread use of the food and herbal remedies in our study population.

Furthermore, in this study, spiritual healing was used by a significant proportion of T2DM patients (12%). Examples of practices reported included prayers, lighting candles in churches and consulting with religious authorities. It is important to note that, in this study, spiritual healing, although it undeniably included the action of praying, was distinct from private, devotional communication (which may include daily petitions for health, blessings, forgiveness, and grace). Inexpensive and easy to use, these therapies have the advantage of being safe [43] with this type of CAM, though health benefits are not guaranteed; there appears to be positive association between religiousness/spirituality, and higher wellbeing and positive effect, as well as a negative association with depressive and anxiety symptoms [44].

Study findings indicated that only 7% of T2DM patients were referred to use CAM by their health practitioners, compared to two thirds of CAM users being referred or encouraged to use CAM by a friend. In addition, only 6.9% informed their physician of such use, despite the fact that patient were recruited from physician’s clinics. Other studies revealed a similar or even lower pattern of reporting [35,36,45-48]. These findings suggest that health care practitioners played a marginal role in regards to their patients’ use of CAM therapies and that they remain largely blinded to their patients’ use of CAM despite the fact that some patients are using CAM on an alternative basis to current treatment. Such a finding is disconcerting since a considerable proportion of CAM users either found CAM therapies ineffective (35.4%) or experienced at least one side effect (10.2%).

The fact that T2DM patients attending public hospitals (usually patients belonging to lower socioeconomic status-SES) and those reporting no health insurance coverage had a higher probability of using CAM therapies reflect a socio-economic discrepancy in regards to CAM use among surveyed patients. Furthermore, study findings indicated that the propensity of using CAM on an alternative basis was associated with lack of insurance. Those patients might have been encouraged to use CAM due to the low cost of such therapies as compared to conventional ones. Similar to our study, use of complementary medication by patients with T2DM in Mexico was correlated with lack of insurance [49]. However, the literature on the relationship between the use of CAM and SES is inconclusive, with a clear geographical discrepancy. For example, a higher SES was correlated with a greater CAM usage in most regions of North America and Western Europe. In contrast, lower SES was associated with CAM usage in countries like Turkey and Hawaii [19]. While, the findings of our study in regards relationship between SES and CAM use are more in synchrony with countries closer to Lebanon (e.g. Turkey), future studies are needed to confirm whether patients are more prone to use CAM as a cheaper alternative to conventional therapies or perhaps due to other environmental and cultural reasons.

In addition to public hospital patients and those with no health insurance, attention should be dedicated to CAM use patterns of patients with longer duration of T2DM and to those reporting the presence of diabetes complications. These patients may resort to using CAM therapies on an alternative or complementary basis due to their fatigue and despair with conventional therapies, to save costs or to try something new Other studies on CAM use among T2DM have also found similar findings [17,19,20,22]. Furthermore, patients seek more alternative therapies because these therapies seem less authoritarian and more empowering and as offering them more personal autonomy [50].

A number of shortcomings in this study are worth mentioning. First, and most importantly, the data collection involved patients waiting in the clinics of two hospitals. Although patients were asked to report their habits and opinions and were assured the confidentiality and privacy of their responses, it cannot be ascertained that patients did not experience the social desirability bias, potentially altering their answers to satisfy their health care providers. Second, and related to the aforementioned point, the external validity of the findings of the current study might only be applicable to T2DM patients attending diabetic clinics. The prevalence and patterns of CAM use in patients that are not regularly being followed up by physicians and those with uncontrolled T2DM might need to be established in a different study design. Third, recall bias might have been experienced by some patients especially that many of them were old and were asked about their recent and life time use of CAM therapies. Finally, although data collection involved well-trained interviewers, it cannot be assured that patients were able to comprehend all the questions in the survey instrument.

### Policy and practice recommendations

The strength of the current study is in it being the first of its kind in Lebanon to examine the prevalence, socio-demographic correlates and mode of CAM use among T2DM in Lebanon. The scale of the study may not reflect the national scene and the need for additional studies of a larger scale is clearly noted. Nevertheless, the attention of concerned stakeholders are attracted to a number of potential policy and practice implications that deem their attention, Such stakeholders include the Ministry of Public Health (MOPH), Syndicates and Orders, medical, nursing and health related schools and research institutions.

Prompted by the findings of this study, the Ministry of Public Health (MOPH) could act on three fronts: First, the concerns in regards to the unsafe use of CAM therapies by T2DM patients and associated side effects need to be thoroughly investigated and regulatory tools need to be utilized in order to protect the consumers. Such tools may range from educating consumers on safe use to the withdrawal of unsafe drugs from the market [51,52]. Second, the study prompts the MOPH on the need to examine the equitable access by all T2DM patients, irrespective of their socioeconomic status, by enhancing the availability and affordability of diabetes care and therapies [53,54]. This could be done through restructuring financing mechanisms, particularly for essential medicines used by T2DM patients, in order to reduce out-of-pocket expenditures [53]. Other potential policy options include prioritized budgeting, access to and promotion of generic medicines, price regulation, improved procurement efficiency [55,56]. Third, to build on the findings of this study and to contextualize and enhance the validity of the recommendations, the MOPH could initiate a national dialogue bringing together all stakeholders (orders, syndicates, medical schools, nursing colleges, media, drug importers, consumer protection agencies, etc.) to discuss enhancing the safe use of CAM therapies with the public in general and diabetic patients in particular [51,57].

The findings of this study also encourage medical syndicates and orders to play examine their role in enhancing both the healthcare providers’, as well as the consumers’ awareness on the safe use of CAM therapies in general and those targeting diabetic patients in particular. Practicing clinicians, nurses, pharmacists and dieticians may need to enhance their awareness not only on available CAM therapies for diabetic patients and their associated advantages and disadvantages, but also on effective communication with their patients in order to encourage them to acknowledge and discuss safe use of CAM therapies with their providers [22,51,52]. Examples of such programs include training programs for patient-centered care and communication skills, initiatives to overcome physicians’ attitudinal barriers toward CAM, professional development programs aiming at educating practicing professionals on the safe and effective use of CAM products [51,58-60]. Linking such programs to continuing education credits could encourage providers to enhance their knowledge and skills about the topic. As the study revealed, particular attention ought to be dedicated to patients attending public hospitals and those that do not have insurance coverage. Syndicates and orders could also join efforts to enhance the public awareness on the safe consumption of CAM therapies by conducting awareness campaigns, annual inventories of approved and safe products published both in print and electronically or by assigning a hotline to offer consumers information on safe use of CAM products [51].

While continuing education programs would potentially enhance the knowledge and skills of current practicing professionals on the safe use of CAM therapies, the examination of medical, nursing and other health related curricula would ensure the future graduating health providers are well educated to discuss the safe use of CAM therapy options with their patients in general and diabetic ones in particular [11,51,52,58,61]. Findings of this study call on medical, nursing and other health related schools to re-examine their curricula integrating CAM education and the means to incorporate such popular therapies with conventional treatments.

Research institutions could also play an important role in investigating the enablers and barriers for safe CAM use among diabetics and the means to enhance this safety. Although this study was able to shed light on a number of important facts and figures related to the prevalence and modes of CAM use among T2DM patients, it is limited in terms of the external validity of its findings and it also had left a number of important unanswered questions. For example, future research studies could build on the findings of this study by examining T2DM patients’ prevalence and determinants of CAM use at the national and regional levels. Studies could also examine the underlying causes for the lack of patients’ reporting of CAM use to their care providers and the strategies to enhance transparency between patients and providers. The regulatory mechanisms and policy instruments that the government could utilize to regulate the CAM market, taking into consideration the Lebanese health care context, could also be examined.

## Conclusion

The use of CAM therapies among T2DM patients in Lebanon is prevalent. Decision makers and care providers are encouraged to consider the potential risks and benefits of CAM therapies, especially in the light of the significant proportion of patients who were found to use CAM on an alternative basis. Particular attention must be dedicated to educating T2DM patients, especially those with a family history of diabetes and those who had the disease for a long period, about the safe use of CAM and the importance to disclose the CAM use to their treating physician. A concerted effort by the government, orders and syndicates, medical, nursing and health schools and educational institutions is required to enhance the safe use of CAM therapies by T2DM patients and to help integrate CAM therapies into the conventional treatment of diabetic patients. A study of a national scale validating the findings of this manuscript is highly recommended.

## Competing interests

The authors declare that they have no competing interests.

## Authors’ contributions

FN contributed to the conceptualization of the study, supervised the data collection and analysis processes and wrote the first draft of the manuscript. DM contributed to the conceptualization of the study, led the data collection and analysis processes and contributed to the manuscript right up. MA contributed to the conceptualization of the study, co-supervised the data collection and analysis processes and played a key role in the write up and submission of the manuscript. HS led the statistical analysis carried out in this study and contributed to the write up. LI contributed to the statistical analysis and contributed to write up. YM contributed to the write up of the manuscript. All authors read and approved the final manuscript.

## Pre-publication history

The pre-publication history for this paper can be accessed here:

http://www.biomedcentral.com/1472-6882/14/185/prepub
